# Real-Time Shape Estimation for Concentric Tube Continuum Robots with a Single Force/Torque Sensor

**DOI:** 10.3389/frobt.2021.734033

**Published:** 2021-10-04

**Authors:** Heiko Donat, Jiecong Gu, Jochen J. Steil

**Affiliations:** Institute for Robotics and Process Control, TU Braunschweig, Braunschweig, Germany

**Keywords:** shape sensing, discrete kirchoff rod, real-time, concentric tube continuum robot, shape estimation

## Abstract

Shape-sensing in real-time is a key requirement for the development of advanced algorithms for concentric tube continuum robots when safe interaction with the environment is important e.g., for path planning, advanced control, and human-machine interaction. We propose a real-time shape-estimation algorithm for concentric tube continuum robots based on the force-torque information measured at the tubes’ basis. It extends a shape estimation algorithm for elastic rods based on discrete Kirchhoff rod theory. For simplicity and efficiency of calculation, we combine it with a model under piece-wise constant curvature assumption, in which we model a concentric tube continuum robot as a combination of segments of planar constant curvatures lying on different equilibrium planes. We evaluate our approach for a single and two combined additively manufactured tubes and achieve an estimation frequency of 333 Hz for two combined tubes with a mean deviation along the backbone of the tubes of 1.91–5.22 mm.

## 1 Introduction and Related Work

**FIGURE 1:**
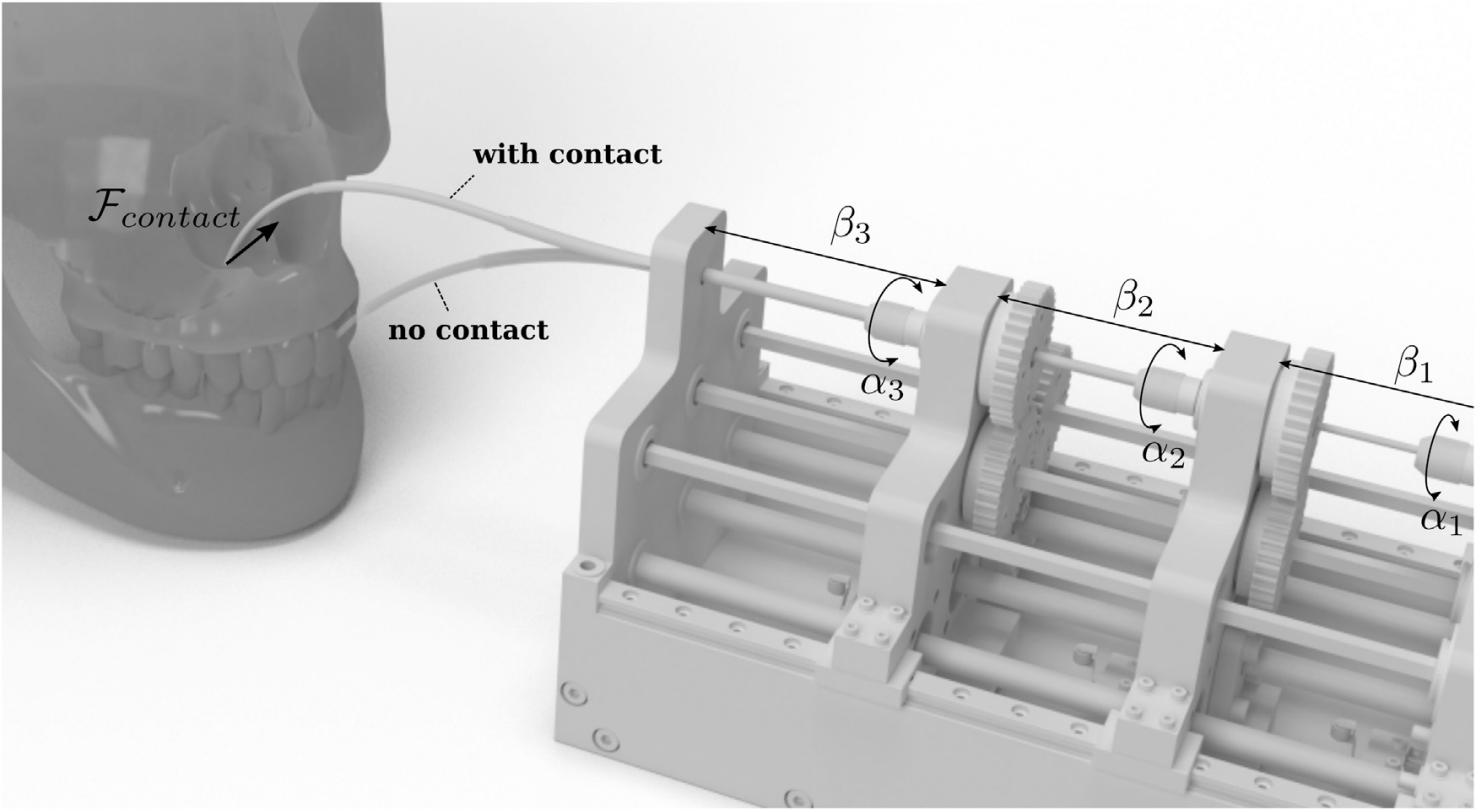
CTRs are especially interesting for medical applications. A major challenge in this area is shape and force estimation. The figure shows a typical actuation unit with tubes without external forces (faded tubes) and in a contact situation. Calculating the change in the tubes’ shape due to those contacts either by direct measuring or estimation is the main task of shape sensing and shape estimation, respectively.

Concentric Tube Continuum Robots (CTRs) are among the smallest and most flexible continuum robots, whose development is interesting for medical applications specifically for surgery. They comprise several super-elastic, pre-curved tubes, which are fit into each other and can be moved by an external actuator system (cf. Figure 1). The tubes are made from shape memory material e.g., Nitinol (NiTi) alloy or other elastic materials such as nylon (Amanov et al., 2015; Morimoto and Okamura, 2016). Considering their dexterity, tracking of the robot’s shape, so-called shape sensing, is important when the robot navigates through critical parts of the human anatomy, e.g., in neurosurgery where damaging surrounding tissue can be dangerous or life-threatening.

The development of dedicated sensors for real-time shape-sensing has been the focus of research in recent years. A promising method for shape-sensing for CTRs is the application of Fiber Bragg gratings (FBG)–based optical sensors. These are specialized optical sensors that are written onto a short segment of an optical fiber, which reflects only a narrow range of wavelengths and transmits all other ranges (Shi et al., 2017). Form shifts create changes in the light wavelengths, the strain on the sensor can be measured, and the associated curvature calculated from which the shape can be modeled.

Due to the small dimensions of FBG-sensors, they can be attached to the CTRs for the purpose of shape sensing. Park et al. (2010) introduced the first FBG-sensor for MRI-compatible biopsy needles embedding three optical fibers with two FBG sensor array nodes, where a mean tip deflection error of 0.38 mm was achieved. Since then research focused on reducing the measuring error by increasing the number of embedded fibers (Shi et al., 2017). Most approaches using FBG-sensors are focusing on the measurement of curvature and bending forces. Recently, (Xu et al., 2016), arranged the optical fibers in a helical shape, supporting the measurements of curvature, torsion, and force, simultaneously. They achieved a root mean squared error of 2–4*%* for curvature and torsion measurements.

While the working channel remains free for the surgical instruments, the embedding of FBG sensors is challenging and manufacturing difficult (Shi et al., 2017). Furthermore, FBGs currently are still costly (da Veiga et al., 2020).

A different option for shape sensing is to use electromagnetic tracking systems (EM) (Franz et al., 2014). EM-tracking systems either provide small sensors to be put into a traceable object or track smaller magnets on the object with an external sensor. With EM-tracking, it is possible to identify single points along the backbone of a tube in real-time (Xu et al., 2013). The real-time capability of EM-tracking enables robot control e.g., stiffness control (Mahvash and Dupont, 2011). However, external magnetic field distortions can compromise EM-tracking leading to larger positioning errors and they have a limited workspace (Shi et al., 2017). In the case of a single sensor and multiple transmitters, tracking has been combined with other model-based methods to reduce the number of traceable points (Wang et al., 2017). A further disadvantage is that the scarce space of the narrow working channel of the CTR is occupied.

An alternative to these approaches is vision-based methods for shape estimation that use image processing. These can be employed without kinematic modeling and hardware modifications for the reconstruction of the shape (Shi et al., 2017). For instance, fluoroscopy was used by Burgner et al. (2011) to reconstruct the shape of CTRs. Using an algorithm for automatic shape segmentation, the centerline of the robot was extracted from two orthogonal views. The shape was then estimated by correspondence analysis. While this method works well and gives a mean error of 0.473 ± 0.353 mm, it requires biplane fluoroscopy systems, which are expensive and require a high radiation dose. In (Lobaton et al., 2013), a new shape reconstruction method was developed based on monoplane fluoroscopy systems by combining information from the kinematic modeling with 2D features. The method achieved a less accurate reconstruction with a mean error of about 0.8 mm. A significant disadvantage of all X-ray procedures is that the patients are exposed to ionizing radiation, potentially leading to cancer or other health problems (Miller, 2009). To avoid such effects, alternative medical imaging techniques for shape recognition have been investigated that involve little or no exposure to radiation, such as ultrasound or magnetic resonance imaging (Ren and Dupont, 2012). Further, common monocular cameras such as endoscopes or microscopes have been investigated as imaging techniques, which have been used to estimate pose information for e.g. micro-stent delivery (Wei and Simaan, 2012).

Besides the advanced technologies discussed above, methods for the application of classical sensors such as Force/Torque-sensor (F/T-sensors) have been developed for the shape estimation of CTRs. In (Xu and Patel, 2012), 2 F/T-sensors were integrated into an actuation unit of a two tube CTR for estimating the tip position. The F/T-sensors were attached to the proximal end of the tubes to measure forces and moments during operation. The sensory information was used to speed up the solution for an initial value problem of a mechanics-based model using Cosserat rod theory. They achieved a position error of less than 3*%* w.r.t. the tube’s length. Xu et al. (2013) further showed an application in real-time control for a CTR under load.

Furthermore, F/T-sensors have been used in several studies to estimate the whole shape of a single elastic rod. Atsushi Yamada et al. (2007) proposed a simple but physically meaningful algorithm for calculating the three-dimensional shape of an elastic rod. The algorithm was further developed, validated, improved, and optimized (Mochiyama, 2016; Takano et al., 2017). Later, Takano and Nakagawa (Nakagawa and Mochiyama, 2018) extended the algorithm to consider the gravitational effects on a rod.

In this work, we extend the analytical model of Nakagawa and Mochiyama (2018) to enable the shape estimation for multiple pre-curved tubes. We consider in addition to the forces and torques at the tubes’ basis their configuration and the effect of superposed pre-curvatures. Our contribution in this work is threefold:

• We show how to include the pre-curvature of the tubes into calculation, without the need to solve a system of ordinary differential equations (ODEs) (Section 2.1)
• Our approach extends the algorithm of Nakagawa to CTRs enabling occlusion-free and real-time shape estimation (Section 2.2).
• We evaluate our approach in two real scenarios for a single tube (Section 3.2) and multiple tubes (Section 3.3) and additionally show the real-time capabilities of our algorithm (Section 3.4).

For this work, we make three modeling assumptions:

• We assume that tubes do not twist relative to one another independent of their configuration.
• The stiffness of the collection of tubes is independent to the configuration.
• Only a single external force is applied to the tubes.

## 2 Proposed Method

A common approach for modeling is to consider the tubes of a CTR as elastic rods, whose static and dynamic behavior can be described by Cosserat rod theory, more precisely Kirchoff rod theory for non-extensible rods. For a rod with one end fixed and the other end free, the shape can be described by four ordinary differential equations (ODEs), which can be solved using a measured wrench at the fixed end of the rod as the initial value. This inital value problem can be solved if only internal forces and moments are given. Applying external forces and moments can be modeled by creating a boundary value problem (Rucker et al., 2010). Both IVP as well as BVP requires numerical integration, which can be applied in real-time, but varies in precision depending on the applied algorithm and the available calculation time. Further, the solution requires a couple of steps, which can end in an error as the solver could not find a solution for the results. This poses a problem for use in hard real-time applications.

The solution’s complexity can be reduced for a single straight tube, where a result can always be given after a fixed time span, by modeling the kinematics analytically, applying discrete Kirchoff rod theory and introducing compliant joints to the resulting chain of rigid links as shown by Takano et al. (2017) and Nakagawa and Mochiyama (2018). The estimation of the tube’s shape becomes a recursive computation along the chain consisting of *n* links, which results in an algorithm with a time complexity of O(n) . In the following section, we show how this idea can be extended towards tubes with pre-curvature and to multiple concentric tubes. Note that we focus on the application towards concentric tubes and restrict the force impact on the tubes to a single location. This limits the applicability towards medical applications where only a single force at e.g. the tip can be considered (Wei and Simaan, 2012) or due to the operational procedure free space is given (Autorino et al., 2013; da Veiga et al., 2020).

### 2.1 Shape Sensing for Concentric Tube Continuum Robots With Precurved Tubes

Nakagawa and Mochiyama’s shape estimation algorithm can theoretically be used for CTRs, since the elastic tubes are also modeled as Kirchhoff rods in kinematic modeling. However, pre-curvature is present even without applied forces and torques. We first show how the pre-curvature of tubes can be considered.

In order to reconstruct the shape of the CTR from measured wrenches at the base with Nakagawa and Mochiyama’s algorithm, we consider the undeformed robot’s backbone as a single discrete kinematic chain, which comprises a series of links and joints. Starting from the base that is fixed to a F/T-sensor, Mochiyama et al. number the joints from 0 to *n* − 1. In contrast to that, we assume the tube consists of *m* segments, such that we number the joints for segment *j* from 0 to *n*_*j*_ − 1. On each joint, i.e. on joint *k*, a local coordinate system *R*_*k*_ ∈ *SO*(3) is attached to joint *k* with its *z*-axis coinciding with the central axis of the previous link. An exception is the base joint, whose coordinate system is the same as the origin. Virtual torsion springs are assigned to each axis of the joint coordinate system. If external loads are present, the torques provided by those virtual torsion springs can be seen as a discrete frame-invariant representation of the internal moments along the rod and can be calculated with the discrete Euler-equation (Yamada et al., 2007; Takano et al., 2017) to further determine the rotational movement of each joint parametrised by Tait-Bryan angles:

$$\theta_{k}={\left(\begin{array}{ccc} \theta_{k,z} & \theta_{k,y} & \theta_{k,x} \end{array}\right)}^{T}\in R^{3}.$$ (1)

We represent the tubes in our shape estimation algorithm as a combination of segments with constant curvature, where each segment *j* lies on a different equilibrium plane. This is a common approach for modeling the CTR’s kinematics (Robert J. Webster and Jones, 2010). Note that straight segments of the tubes are described by a curvature of 0 mm^−1^.

**FIGURE 2:**
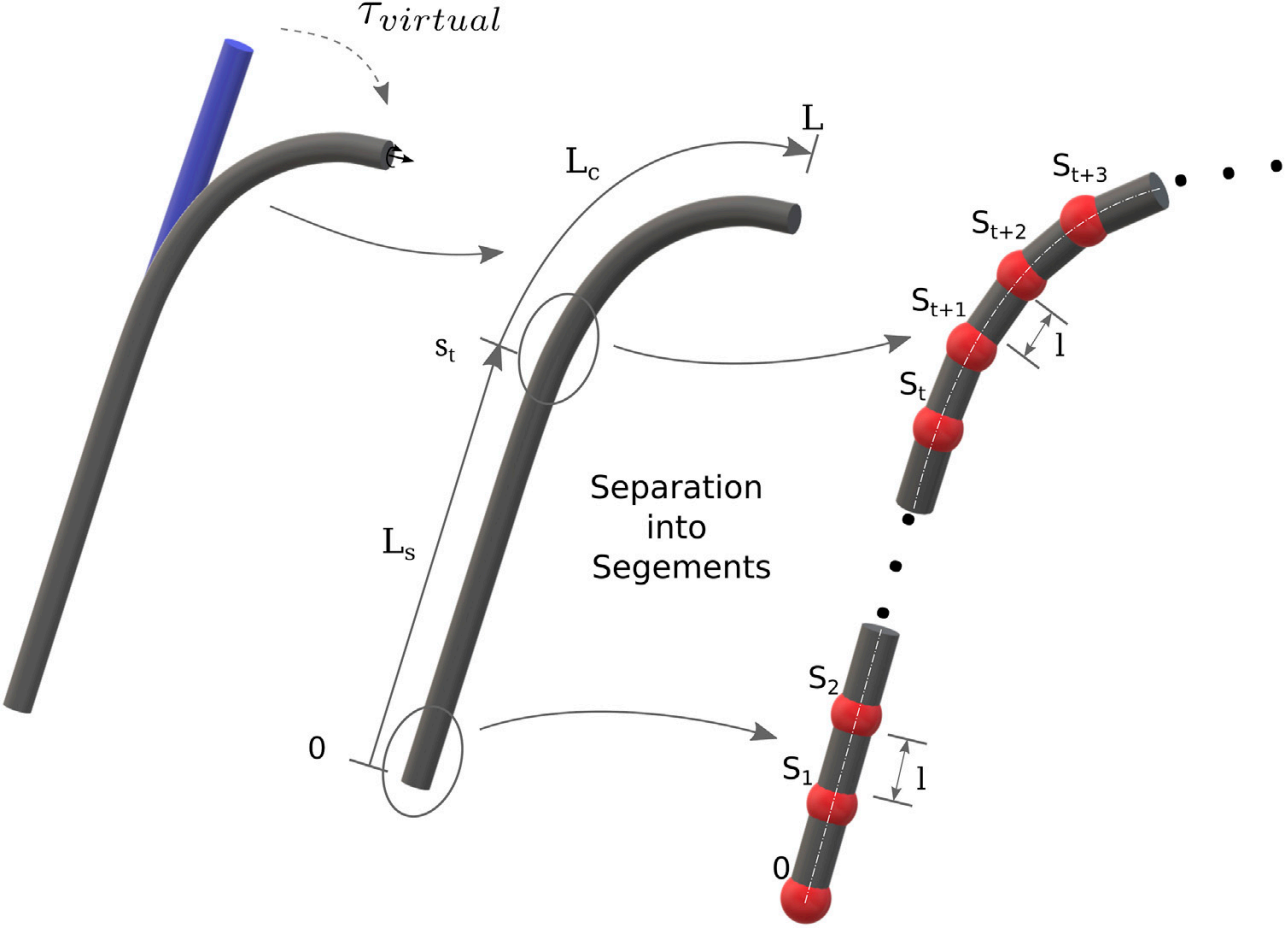
Initially, all tubes (also nested) are considered as straight and the pre-curvature is accounted as virtual loads. Each tube is divided into a straight and a curved segments. The *m* segments are discretized into *n* sections of length *l*.

We aim to find the initial configuration of the kinematic chain, described by joint angles $\theta_{k}^{*}$ and local rotation axes $a_{k}^{*}$ at each joint, by approximating the constant curvatures of each segment *j* of a tube *i* as the angular rate of change of the local Frenet frame (Robert J. Webster and Jones (2010)) with respect to the arc-length *s*. Throughout this work we consider, without loss of generality, that pre-curvature is applied locally around the *y*-axis of a segment:

$$u^{*}\left(s\right)={\left(R^{T}\left(s\right)\dot{R}\left(s\right)\right)}^{⊕}={\left(\begin{array}{ccc} 0 & \kappa_{y} & 0 \end{array}\right)}^{T},$$ (2)

where ⊕ is the conversion operator for an element of *so* (3) to its corresponding element in $R^{3}$ . Instead of discretizing the curvature using osculating circles for each segment (Bobenko et al., 2008), we discretize the tubes into a link chain with an equal link length of *l*. In our approach, we represent pre-curvature as virtual external loads which deform an originally straight elastic rod to its pre-curved state (cf. Figure 2). We approximate the deformed shape, using an initially straight discrete serial chain, by assuming the energy stored in the virtual torsion springs along the *y*-axis of the *k*th local joint coordinate system (Mochiyama, 2016) is equal to the energy stored in a deformed segment section between *kl* and (*k* + 1)*l*. Since we assume a planar pre-curvature, the local curvature indicates that each segment of the robot only bends about the local *y*-axis, such that the joint angles and rotation axes can be obtained by solving the following equation:

$$\frac{1}{2}\int_{kl}^{\left(k+1\right)l}u^{*}{\left(s\right)}^{T}Ku^{*}\left(s\right)\;ds=\frac{1}{2}\xi_{j,y}\theta_{k}^{*2},$$ (3)

where $K=diag\left(\begin{array}{ccc} E_{i}I_{i} & E_{i}I_{i} & G_{i}J_{i} \end{array}\right)$ is the stiffness matrix constituted of stiffness of the *j*-th segment, describe the initial joint angles of the kinematic chain and *ξ*_*j*,*y*_ is the spring constant for each segment section. Note, in the case of constant pre-curvature, the calculation of the forming energy simplifies to: $E=\frac{1}{2}lu_{j}^{*T}{\mathrm{Ku}}_{j}^{*}$ . By assuming *ξ*_*j*,*y*_ = *E*_*i*_ *I*_*i*_, which is for all joints in the same segment equal based on the constant curvature assumption, and that the curvature is a rotation around the local *y*-axis *e*_*y*_ about *ξ*_*j*_ *l*, we can solve towards $\theta_{k}^{*}$ by canceling *E*_*i*_ *I*_*i*_ on both sides and yield:

$$\theta_{k}^{*}={\mathrm{lu}}_{j}^{*}.$$ (4)

### 2.2 Shape Estimation for Multiple Tubes

We now consider the application with multiple tubes. Thus the configuration of the tubes relative to each other must be considered. Note that we make the assumption that the tubes cannot be twisted relative to another. Further, we assume that the stiffness is independent of the configuration. Given the constant curvature assumption, we can apply the CTR-specific arc parameter mapping stated by Robert Webster and Jones (2010). Note, we assume that the local *z*-axis of each frame along the tube coincides with the backbone of the robot. Each tube *i* can be rotated around the *z*-axis of the base by an angle *α*_*i*_. Furthermore, each tube can be extended and retracted, which is described by the parameter *β*_*i*_ ∈ [−*L*, 0], where *β*_*i*_ = 0 describes a fully extended tube. From the manufacturing of the tubes, it is known where each tube’s straight and curved part starts and ends, which can be described as a sub-interval of arc length *s* ∈ [0, 1] parameterizing the tubes’ backbone. Parameterized by *s* each end of a straight or curved part marks the end of a distinct constant curvature segment on the CTR’s backbone.

For each segment *j*, we can determine the pre-curvature *κ*_*j*_ considering that moments will be constant along the segment. Given the $\kappa_{i}^{*}$ which is the pre-curvature of the tube *i*, we can determine the curvature components:

$$\begin{array}{cc} \kappa_{x,j} & =\frac{\sum_{i}E_{i}I_{i}\kappa_{i}^{*}\cos\alpha_{i}}{\sum_{i}E_{i}I_{i}}\\ \kappa_{y,j} & =\frac{\sum_{i}E_{i}I_{i}\kappa_{i}^{*}\sin\alpha_{i}}{\sum_{i}E_{i}I_{i}}, \end{array}$$ (5)

where *E*_*i*_ is the Young’s modulus, *I*_*i*_ the second moments of area about the local x- and *y*-axis, which we assume to be equal, and *α*_*i*_ the rotation around the *z*-axis of the tube *i*. Because all tubes share the same neutral axis and we assume that the material is isotropic, we can sum up their individual stiffness values. Given the projections of the curvatures onto the new plane intersecting the combined tubes, we can calculate the combined curvature:

$$\kappa_{j}=\sqrt{\kappa_{x,j}^{2}+\kappa_{y,j}^{2}}.$$ (6)

The deformed shape of the robot, as well as the position and orientation of the robot tip, can be then calculated with the given base position *p*_0_ and orientation *R*_0_ recursively:

$$\begin{array}{cc} R_{k+1} & =R_{k}Rot\left(e_{y},\kappa_{j}l\right)Rot\left(\theta_{k}\right),\\ p_{k+1} & =p_{k}+lR_{k+1}e_{z}. \end{array}$$ (7)

We follow the work of Nakagawa and Mochiyama (2018) and also consider the influence of the gravitational forces on the tubes. Therefore, for each segment section of length *l*_*j*_ we calculate the mass *M*_*j*_ with respect to the set of tubes I acting in this section:

$$M_{j}=\frac{l_{j}}{L}\overset{I}{\underset{i}{\sum }}M_{i},$$ (8)

where we assume that the weight is uniformly distributed over a tube and *L* is the length of the whole backbone. The calculation for segments is described in pseudo-code in Alg. 1, where $c=\frac{p_{k}+p_{k-1}}{2}$ for the compensation of gravitational forces (Nakagawa and Mochiyama, 2018). All input and output model parameters are listed in Alg. 1, too. Note that the code in Alg. 1 is executed for each segment *j* of the backbone of a CTR. Given *m* segments and *n* sections the overall computational complexity increases to O(m⋅n) where for good accuracy *m* ≪ *n*.

**Algorithm 1:** Shape estimation algorithm for each segement of a CTR with precurved tubes

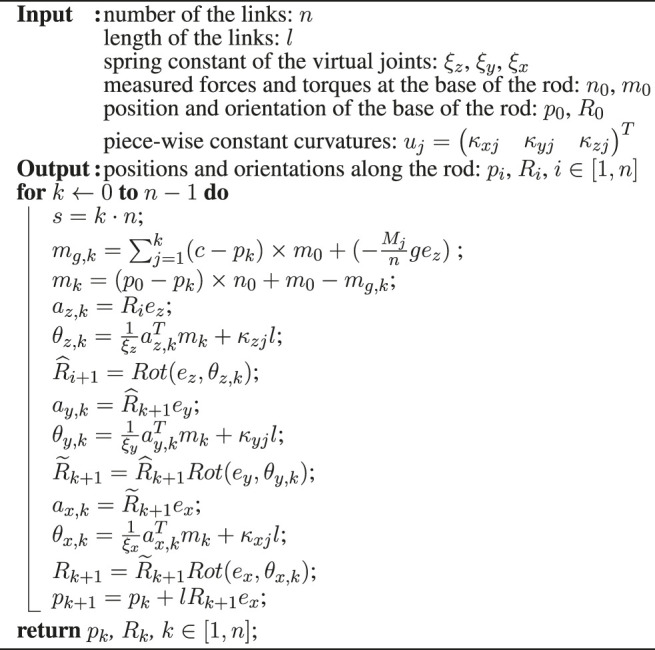

## 3 Evaluation

To validate our proposed algorithm, we investigated two scenarios: (I) an additively manufactured tube directly connected to the flange of a F/T-sensor, and (II) nested tubes with different pre-curvature actuated by a custom-made actuation device.

### 3.1 System and Experiment Setup

**FIGURE 3:**
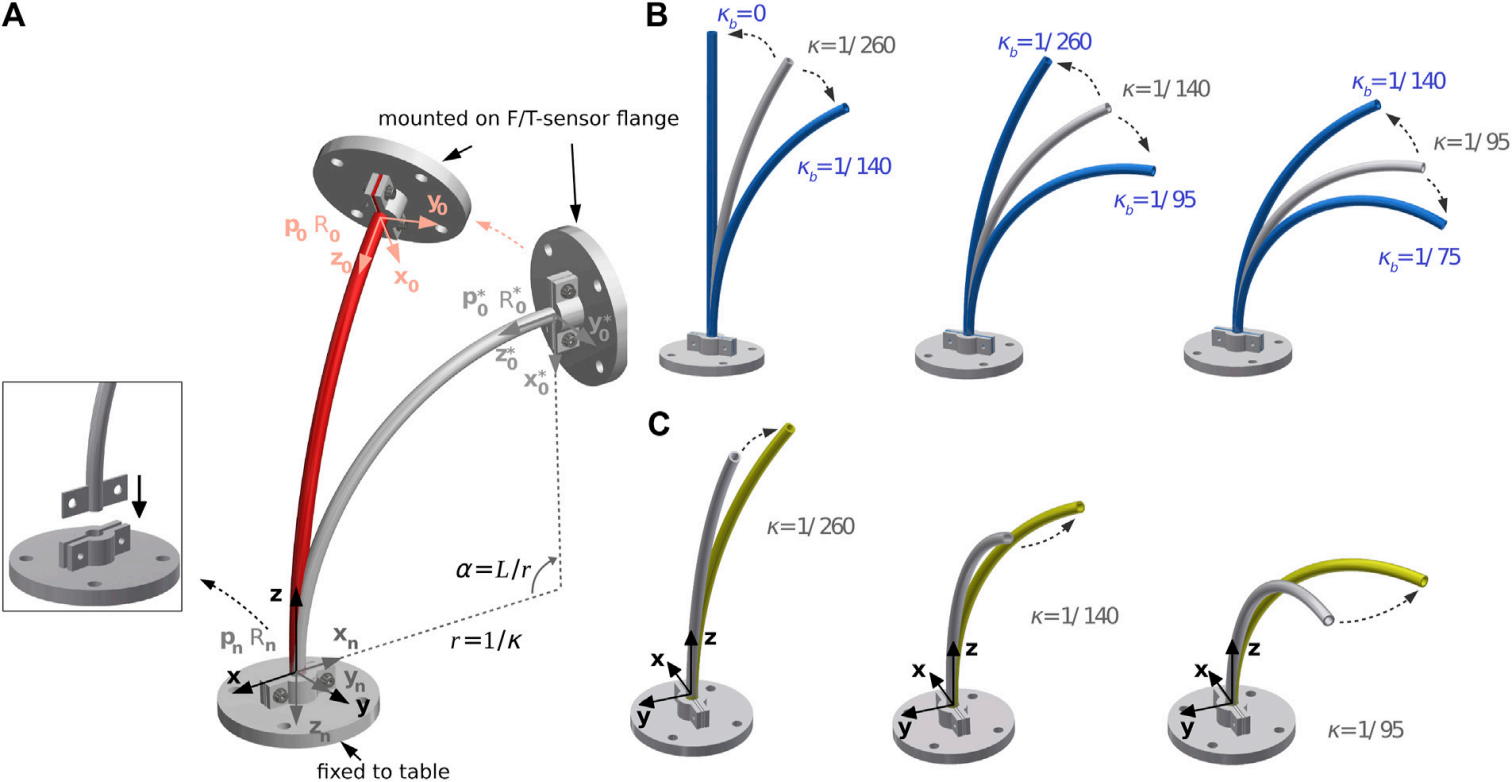
Overview of the bending and torsion test for three single pre-curved tube: **(A)** general setup, **(B)** positional changes for bending, and **(C)** positional changes for torsion.

We used a Kuka LBR iiwa 7 R800 robot (Kuka AG, Germany) for moving the tube with a JR3 F/T-sensor 50M31 A-I25 (JR3, Inc., United States) attached at the flange. Two custom-made 3D-printed clamps fixed the tube to the F/T-sensor and a table (Figure 3A) shows the general setup of the clamps). We printed three 150 mm tubes with a constant curvature of 1/95 mm^−1^, 1/140 mm^−1^ and 1/260 mm^−1^, resembling a bending angles of 90°, 60°, and 30°. Additionally, we printed small belt line hooks at the ends of the tubes to keep them fixed. For the two-tube experiment, we printed one inner tube with a length of 150 mm, and three different outer tubes of length 50 mm, 75 mm, and 100 mm. All tubes have been made of taulman3D 618 Nylon (taulman3D, United States). Although, nylon has a higher degree of plastic deformation it has been shown that CTR made of nylon achieve similar accuracies as robots made of NiTi (Amanov et al., 2015; Morimoto and Okamura, 2016). We chose the inner and outer diameters for the applied tubes such that the plastic deformability is low and the tubes are printable with an Ultimaker3 (Ultimaker, United States). Due to fabrication limitations, it was only possible to produce a rod with a diameter of 2.8 mm. A thin rod was preferred over a thicker one for more elasticity. Further parameters are listed in Table 1, where *L*_*s*_ describes the length of the straight part of the tube and *L*_*c*_ its pre-curved part, respectively. The algorithm was implemented in *Python*3.8 and run on an Intel Core i7-7,700@4.5 GHz PC. Communication with Kuka Sunrise.Os and the F/T-sensor was handled with Robotics Service Bus (RSB) (Wienke and Wrede, 2011) and ROS Melodic Morenia (Quigley et al., 2009).

**TABLE 1:** Parameters of the CTR used in this work.

*i*	*L*_s_	*L*_c_	*κ*	*d*_out_	*d*_in_
1	178.80	150	10.47	2.8	0.0
2	109.3	100	6.98	5.1	3.4
2	84.3	75	6.98	5.1	3.4
2	59.3	50	6.98	5.1	3.4

All values in mm, except *κ* in mm^−1^. Tube i=2 is listed with three different lengths.

### 3.2 Evaluation for One Concentric Tube With Pre-curvature

For the evaluation of our proposed algorithm on a single pre-curved tube, we followed an experiment protocol similar to Nakagawa and Mochiyama (2018) and applied planar deformation and torsional deformation separately, and in combination on the tube. Figure 3A) shows an undeformed tube, where the world coordinate system is fixed in the clamp at the end of the tube and the tube’s base is mounted in the clamp at the F/T-sensor which is mounted at the robot flange. The *z*-axis of the world coordinate system is tangent to the tube centerline and the negative *x*-axis is directed to the center of the osculating circle with radius of $r=\frac{1}{\kappa }$ . The position and orientation of the base of the tube with respect to the world coordinate system is defined as follows:

$$\begin{array}{c} p_{0}=\left(\begin{array}{ccc} -\frac{1-\cos\kappa L}{\kappa } & 0 & \frac{1-\sin\kappa L}{\kappa } \end{array}\right)\\ R_{0}=\left(\begin{array}{ccc} -\cos\kappa L & 0 & \sin\kappa L\\ 0 & 1 & 0\\ -\sin\kappa L & 0 & -\cos\kappa L \end{array}\right) \end{array}$$ (9)

This information is used to transform the calculated shape from the sensor coordinate system to the world coordinate system. The pose of the end of all tubes is set as follows and is compared with the calculated pose:

$$\begin{array}{c} p_{n}=\left(\begin{array}{ccc} 0 & 0 & 0 \end{array}\right)\\ R_{n}=\left(\begin{array}{ccc} -1 & 0 & 0\\ 0 & 1 & 0\\ 0 & 0 & -1 \end{array}\right) \end{array}$$ (10)

Like in the evaluation of the algorithm of Nakagawa the tubes are deformed by bending and torsion. In the following, the different test poses, based on the position and orientation of the base of the tube after deformation *p*_0_ and *R*_0_ are presented.

#### 3.2.1 Planar Deformation

Figure 3B), the three pre-curved tubes are deformed by pure bending to a larger and smaller curvature. The pose of the base of the tube after deformation *p*_0_ and *R*_0_ is defined by the following equations:

$$\begin{array}{c} p_{0}=\left(\begin{array}{ccc} -\frac{1-\cos\kappa_{b}L}{\kappa_{b}} & 0 & \frac{1-\sin\kappa_{b}L}{\kappa_{b}} \end{array}\right)\\ R_{0}=\left(\begin{array}{ccc} -\cos\kappa_{b}L & 0 & \sin\kappa_{b}L\\ 0 & 1 & 0\\ -\sin\kappa_{b}L & 0 & -\cos\kappa_{b}L \end{array}\right) \end{array}$$ (11)

The curvatures *κ*_*b*_, which can be seen in Figure 3B, is set such that the angle of curvature of the tube to be deformed *α* = *L*/*r* is changed by approximately ±30° to avoid plastic deformation (Amanov et al., 2015; Morimoto and Okamura, 2016). In addition, large changes in curvature are uncommon during the operation of the CTRs, and due to the resulting plastic deformations that result, they are also not desirable (Greiner-Petter, 2019).

The tube with a pre-curvature of 1/260 mm^−1^ is made into a quasi-straight shape. The position of the tube base is not calculated with the formula described above, but defined as $p_{0}={\left(\begin{array}{ccc} 0 & 0 & L \end{array}\right)}^{T}$ .

#### 3.2.2 Torsion

As shown in Figure 3C), the tubes are twisted by rotating the coordinate system by *β* = 30°. The pose after the rotation is given by:

$$\begin{array}{c} p_{0}=\left(\begin{array}{ccc} -\frac{\left(1-\cos\kappa L\right)\cos\beta }{\kappa } & -\frac{\left(1-\cos\kappa L\right)\sin\beta }{\kappa } & r\sin\kappa L \end{array}\right)\\ R_{0}=Rot\left(e_{3},\beta \right)\left(\begin{array}{ccc} -\cos\kappa L & 0 & \sin\kappa L\\ 0 & 1 & 0\\ -\sin\kappa L & 0 & -\cos\kappa L \end{array}\right). \end{array}$$ (12)

#### 3.2.3 Combined Deformation

Starting from the poses for planar deformations, rotations around the z- and *x*-axis of the coordinate system are additionally performed at the tube base with an angle of *γ* = 30° each in succession. Thus, an originally planar tube is deformed into a spatial curved tube. The pose after deformation can be given as follows:

$$\begin{array}{c} p_{0}=\left(\begin{array}{ccc} -\frac{1-\cos\kappa L}{\kappa } & 0 & \frac{1-\sin\kappa L}{\kappa } \end{array}\right)\\ R_{0}=\left(\begin{array}{ccc} -\cos\kappa L & 0 & \sin\kappa L\\ 0 & 1 & 0\\ -\sin\kappa L & 0 & -\cos\kappa L \end{array}\right)Rot\left(e_{3},\gamma \right)Rot\left(e_{1},\gamma \right). \end{array}$$ (13)

Due to movement limitation when bending the tube with a pre-curvature of 1/260 mm^−1^ straight, no change around the *x*-axis was possible, and therefore this case was neglected in the evaluation. Altogether we evaluated 14 different deformations.

#### 3.2.4 Simulation Test and Results

**FIGURE 4:**
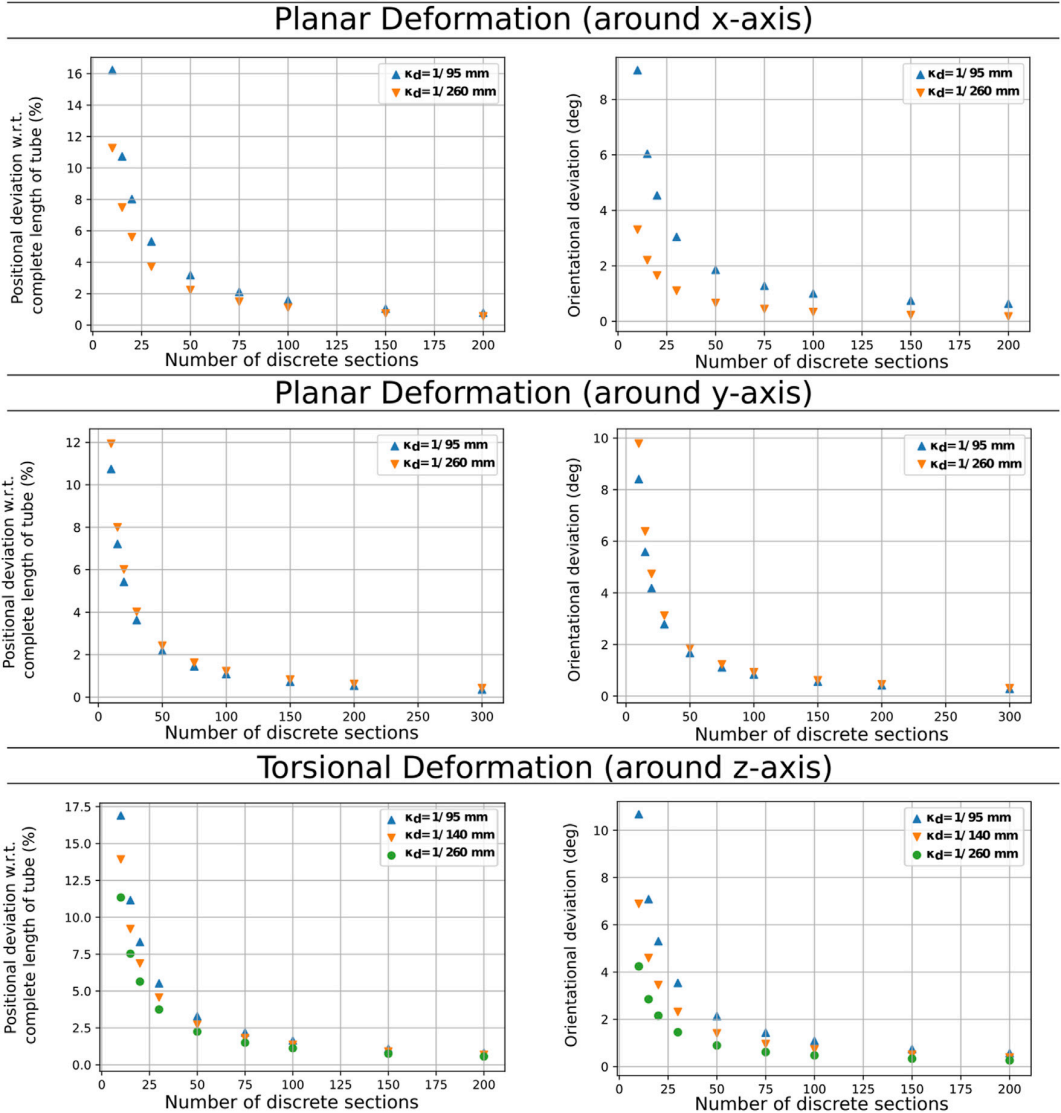
Comparison discretization steps and position deviation for the three different evaluation scenarios for the tube with a pre-curvature of 1/140 mm^−1^.

We validate our approach for the three pre-curved tubes first in simulation by comparing our algorithm’s solutions with positions and orientations given by an implementation of the more precise but more computational costly method of Rucker et al. (2010) to evaluate dependency between segment length and positional and orientation deviation. Figure 4 shows the results for the deviation of tip position and orientation for different discretization resolutions compared to the model by Rucker et al. (2010) relative to the total length for the tube with a pre-curvature of 1/140 mm^−1^. The results show that the position and the orientation error reduce fast when the number of segments increases. For *n* = 50 we achieved a position error of less than 2.7*%* w.r.t. the tube’s length and an orientation error of less than 2°. It is worth noting that the position deviation for the same *n* is almost the same for all pre-curvatures. The orientation deviation in twisting depends on the discretization errors. The additional torsion introduced through planar deformations does not increase the errors. Therefore, we conclude that the accuracy does not depend on the complexity of the deformation.

#### 3.2.5 Experiment Results on the Real Tube

For the evaluation of accuracy, the number of links in the experiment was set to *n* = 50. A comparison of the position and orientation deviation from the reference values was again performed, this time using measurements. A minimum deviation of 2.068 mm (corresponding to 1.4% w.r.t. the tube length) and 1.877° were calculated where the tube was formed into a straight shape from a pre-curvature of 1/260 mm^−1^. The maximum average deviation was obtained when the tube was bent with the pre-curvature of 1/95 mm^−1^, which resulted in 8.783 mm in position and 6.493° in orientation. All results for rotational, torsional, and spatial deformations are listed in Tables 2, 3, and 4, respectively. Given that the tube with a pre-curvature of 1/260 mm^−1^ bent the tube into a straight one, no further spatial movement was possible, such that this data was neglected.

**TABLE 2:** Results of rotational deformation.

*κ*(*mm* ^−1^)	*κ*_*b*_ (*mm* ^−1^)	Mean error	Best result
$\frac{1}{95}$	$\frac{1}{75}$	8.783 mm	7.938 mm
		4.278°	3.073°
	$\frac{1}{140}$	7.438 mm	5.453 mm
		6.493°	4.206°
$\frac{1}{140}$	$\frac{1}{95}$	5.937 mm	5.623 mm
		4.630°	2.785°
	$\frac{1}{260}$	5.778 mm	4.158 mm
		6.123°	4.525°
$\frac{1}{260}$	$\frac{1}{140}$	5.651 mm	4.691 mm
		4.880°	4.252°
	0	3.278 mm	2.068 mm
		2.937°	1.877°

**TABLE 3:** Results of spatial deformation.

*κ*(*mm* ^−1^)	*κ*_*b*_ (*mm* ^−1^)	Mean error	Best result
$\frac{1}{95}$	$\frac{1}{75}$	7.913 mm	7.360 mm
		11.263°	9.755°
	$\frac{1}{140}$	8.715 mm	7.094 mm
		12.210°	10.990°
$\frac{1}{140}$	$\frac{1}{95}$	3.397 mm	2.715 mm
		16.758°	16.535°
	$\frac{1}{260}$	12.740 mm	8.873 mm
		11.608°	9.102°
$\frac{1}{260}$	$\frac{1}{140}$	2.110 mm	1.074 mm
		14.632°	13.842°

**TABLE 4:** Results of torsional deformation.

*κ*(*mm* ^−1^)	Mean error	Best result
$\frac{1}{95}$	10.414 mm	8.865 mm
	5.963°	4.761°
$\frac{1}{140}$	8.664 mm	7.452 mm
	9.762°	9.331°
$\frac{1}{260}$	8.486 mm	7.475 mm
	11.121°	9.172°

Overall, our algorithm can achieve similar results for individual tubes 3D-printed from nylon as Nakagawa and Mochiyama (2018) for steel strips. The average position deviations for spatial deformations were comparable to those for straight rods.

### 3.3 Evaluation for Multiple Pre-curved Tubes

Since fixing the tubes severely restricts the workspace of a CTR and combined tubes cause complex movements when rotated into each other for larger rotations, we chose a different recording scenario for evaluating our algorithm with multiple tubes. Using a motion capture system, the shape of the robot’s tubes is tracked to obtain accurate position measurements. For this purpose, we applied 5 mm wide retro-reflective tape (3M, Germany) to the tubes by attaching 15 markers separated by a distance of 5 mm. These markers were recorded with seven cameras of the motion capture system (Optitrack-Prime-13-System, NaturalPoint, United States) with a mean position error of 0.235 mm.

**FIGURE 5:**
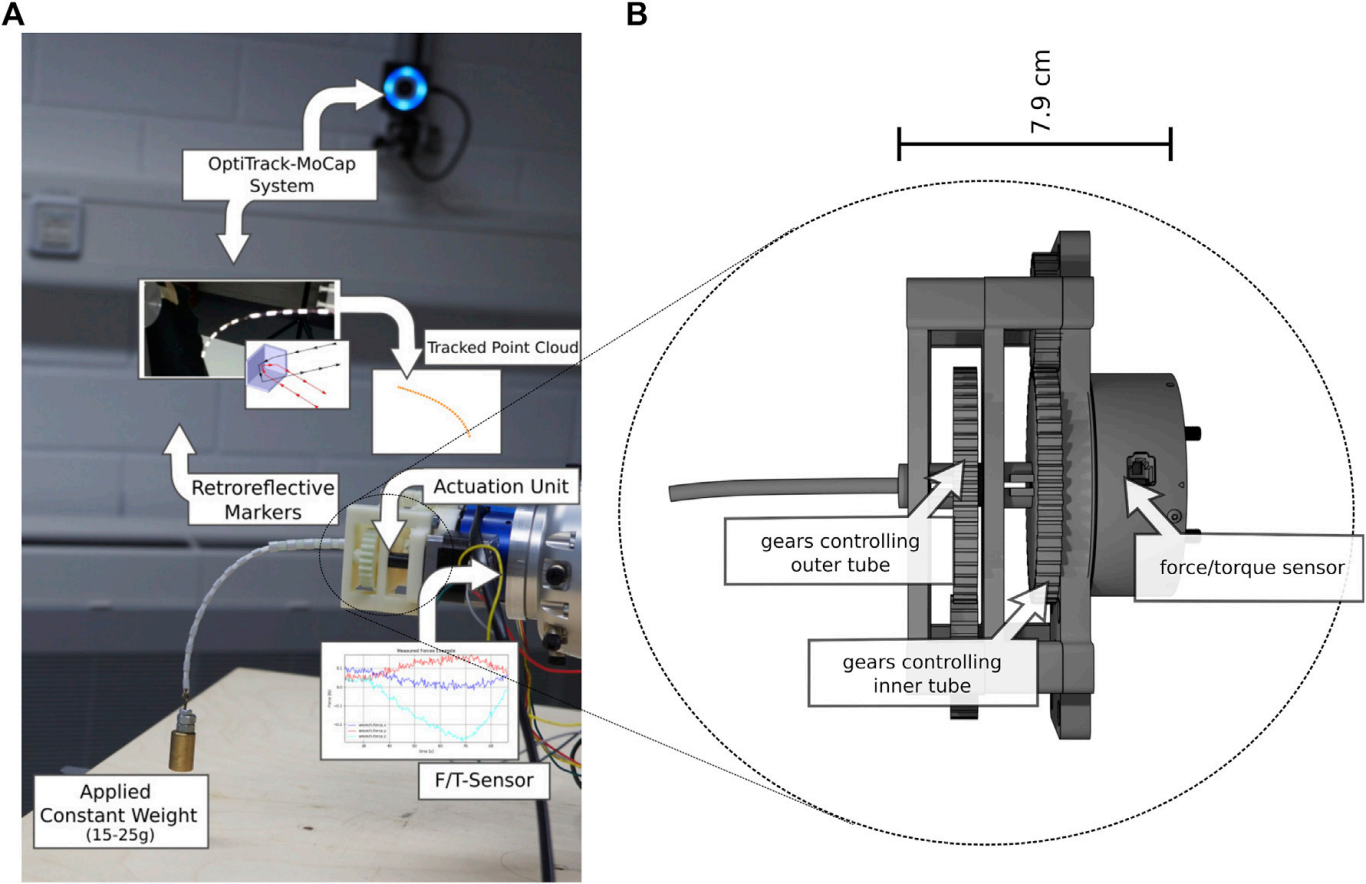
Overview of the recording with labels for the hardware parts and visualization of their recordings. **(A)** Experimental setup **(B)** Schematic of custom made actuation unit with F/T-sensor. Further, a) shows the combination of an inner tube of 150 mm with and outer tube of 50 mm and a weight of 25 g, which yielded large deflections of the tubes.

The printed tubes were rotated using a custom-made drive unit consisting of two stepper motors and a 3D-printed gear box (c.f. Figure 5). Lacking a translation joint in our actuation unit due to the integration of the F/T-sensor, we printed three outer tubes of different lengths as mentioned above to emulate elongation. We assumed that the outer tube can elongate a maximum of 100 mm. As mentioned previously, due to limitations in the additive manufacturing of the small 3D-printed tubes, the inner tube was produced as a nylon rod. An additional ring was printed on its tip to attach two different weights (15 and 25 g) for applying load to the tubes.

We recorded 9,814 data samples without applied loads to an outer tube elongation of 100 mm and a step width of 1° for the tubes’ rotation. Furthermore, we recorded 2,863 data samples with applied loads for the three described outer tube lengths with a step width of 9°. Each single sample recording without, applied loads lasted 0.5 s to reduce the possibility of frictional snapping effects. Each sample recording with applied load to the tubes’ tip lasted 6 s, consisting of 5 s to relax vibrations introduced by the inertia of the applied weight at the end of the tube plus 1 s to measure and average the measured force and torque. The recorded movements were limited to the range [−*π*/2, *π*/2] (rad) for the inner and outer tubes.

The restrictions have been introduced because a rotation over *π*/2 rad did not lead to any significant tip movement due to the applied weight. The resulting torsional forces twisted the tube and snapping effects occurred when overwinding, which represents a special case in the application of CTRs we did not want to investigate. The outer tube performed one step in a positive direction of rotation as soon as the inner tube had reached the angle − *π*/2 or *π*/2. After recording, samples with missing positions along the shaft or additional points due to noise were dropped. The recording setup is shown in Figure 5.

**FIGURE 6:**
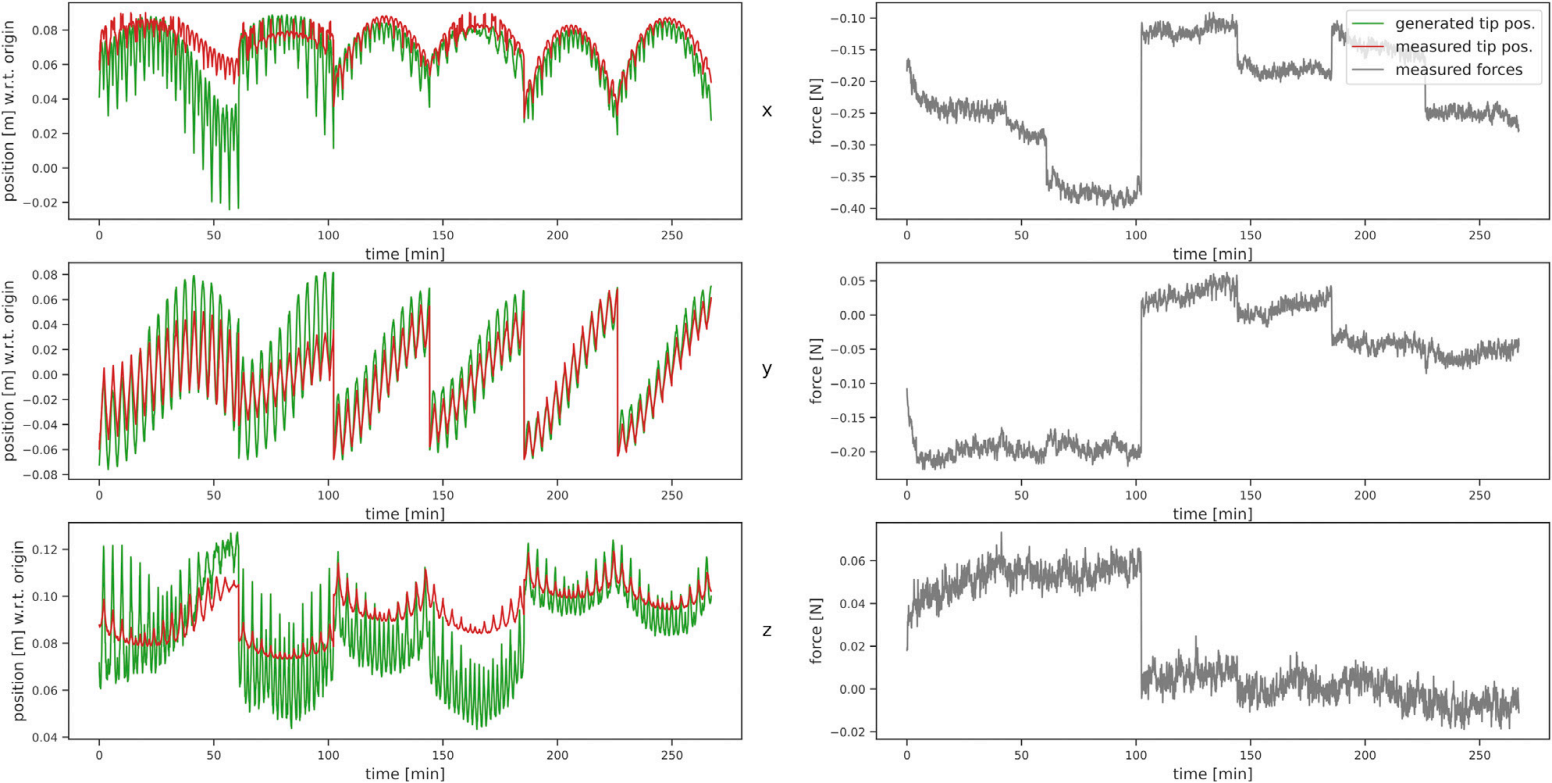
Comparison of measured (red) and estimated (green) tip positions on the left, with the measured force in x-, y-, and z-direction of the base frame on the right, respectively.

Based on the recorded wrenches, we calculated the corresponding virtual positions (positions given by our algorithm derived in Section 2) for each configuration. As discussed above, 15 points were recorded with the tracking system and the same number of virtual points were generated per sample. A comparison of measured and corresponding calculated tip positions with respect to the measured forces is shown in Figure 6. We calculated the maximum absolute error (MAE) between the virtual (model-based calculated) and measured points along the robot shaft and the inner tube’s tip position and calculated the standard deviation (STD) of the error. For the data without loads applied to the robot, we achieved a MAE of 3.63 ± 3.32 mm for all points along the shaft and 1.74 ± 1.98 mm for the tip position deviations. Table 5 shows the results for all six tube-weight combinations. The best tip position MAE of 4.47 ± 2.94 mm and the best MAE of 1.39 ± 1.68 mm accounting for all positions along the backbone was achieved for an outer tube of length 100 mm and 15 g tip load.

**TABLE 5:** Results of multi-tube experiment. All deviations are measured in mm. Column labels describe length of outer tube/weight at tip.

	50 mm/15 g	50 mm/25 g	75 mm/15 g	75 mm/25 g	100 mm/15 g	100 mm/25 g
MAE along backbone	5.22	5.29	2.89	3.69	1.39	1.91
STD along backbone	7.91	8.34	3.76	6.01	1.68	2.43
MAE tip position	16.11	14.74	9.50	14.52	4.47	6.02
STD tip position	12.77	11.95	6.52	11.50	2.94	3.98

In contrast to the results for individual tubes in Section 3.2.5, the results for multiple concentric tubes show larger variances in error. Specifically, for the deviation in *z*-direction our model is overestimating the deflection. This is particularly evident in the results with an outer tube of only 50 mm, where the deviations are in the centimeter range, caused by larger bending due to the weight.

As Takano et al. (2017) state in their work, the approximation of the bending by the discrete Euler-equation produces larger deviations to the actual curvature, if the tubes experience a strong bending. In addition to that, larger deflection as given in this evaluation lead to relative movement between the tubes, which we do not consider in our model. Note that such large deflections are not commonly assumed for tubes with such stiffness. Still, a solution should be found in further investigations, however, this was not the main focus of this work and the tests with larger deflection show the limitation to our approach. Further, smaller factors for the deviations occur partially due to the evaluation setting, in which the greater weight of the actuation unit generates an additional force, which is substracted out, but still affects the sensor readings. Furthermore, the JR3 FT-sensor as displayed in Figure 5 has a slight coupling between the force and torque axes, which increases the measurement error. Another minor factor is the inaccuracy in the determination of the material properties. We assume a symmetrical cross-section, which, however, is an idealization and not possible to be achieved perfectly with the applied printing process. The disadvantage of dependence against sensor data quality and well chosen model parameters is also mentioned by Takano et al. (2017).

Since we calculate the positions along the backbone by *m* ⋅ *n* discrete transformations the errors along the tubes add up. It can be seen that the deviation towards the tip of the tubes becomes more and more pronounced, which can be explained by the structure of the discretized model, which accumulates errors through the iterative calculation from the basis to the tip. This is, however, inevitable, as we rely on a measurement at the basis.

Continuous models achieve better results when large deflections are present and tip position and orientation is given. However this information is not always available and the proposed approach can estimate the shape solely based on the wrench information at the basis. Furthermore, the results have a favorable trade-off between good accuracy at highly efficient computation as the algorithm always produces the same result in the same duration. It makes the approach applicable for hard real-time applications, where cycle deadlines always need to be met, and which are necessary for ensuring the safety of medical applications.

### 3.4 Real-Time Capability

To evaluate our assumption about real-time application, we evaluated the computation time of the algorithm, which depends on the number of links, i.e. the number of computational steps required. For *n* = 50, an average computation time of less than 3 *ms* was measured, which enables an estimation frequency of ca. 333 Hz. This is sufficiently fast for real-time shape estimation. However, if the sensor is noisy, a filter is needed to improve the shape estimation results. In this work, a fifth-order Butterworth low-pass filter was used to suppress noise. This delays the calculation, but still a shape estimation with 20 Hz can be achieved which can be hardware optimized in potential real applications. Given that the algorithm always produces a response after *n* ⋅ *m* calculation steps, we concluded that hard real-time deadlines for control can be met.

## 4 Conclusion

In this work, we introduced an algorithm for shape estimation of CTRs which is applicable for real-time applications. It extends the algorithm of Nakagawa and Mochiyama (2018) for pre-curved tubes combining it with constant curvature modeling to achieve the application in multiple concentric tube settings. Pre-curvature is represented as virtual torques applied on the virtual joints of the discrete kinematic chain which models a CTR’s backbone. Furthermore, to enable the application with CTRs, we extend the algorithm towards multiple tubes of a robot, using the constant curvature assumptions for the segments of the tubes.

In comparison to conventional approaches, which compute the shape of a CTR as a system of ODEs and apply numerical integration, our approach is based on recursively applied linear operations. The robot is modeled as *m* segments, where each segment is described as a discrete chain of *n* links, which yields the computation complexity of O(m⋅n) , where *m* ≪ *n*. Our discretization of the *m* segments under the assumption of constant curvature into equal-length sections simplifies the modeling and in combination with the representation of pre-curvature as virtual torques preserves the joint and tip orientation well.

We evaluated our approach with two experimental setups and obtained similar results as the referenced literature with an estimation frequency of up to 333 Hz. The current measurements are based on a *Python* 3.8 implementation, which limits the cycle time due to the weak performance of *Python* in loop executions. In future work, we plan to realize the algorithm in a compiled language.

A limitation of this proposed discretization method occurs when a larger deformation is applied to the tubes, as seen in the evaluation for multiple tubes, which leads to greater position errors. This is caused by the discrete Euler-equation, the not modeled relative motion of the tubes and in a minor case by the dependency on well determined model parameters.

Although the accuracy is lower for large deflections in comparison to the referenced methods the algorithm only depends on the signal of a single conventional F/T-sensor, which renders it as a cost-efficient alternative to e.g., FBGs. Furthermore, the algorithm never exceeds O(m⋅n) calculation steps, which makes the algorithm useful for application in hard real-time scenarios. In future work, we want to combine the algorithm with monocular vision-based methods to enhance the vision information with kinematic information given by our algorithm. Further, we aim to combine the proposed algorithm with real-time capable machine learning techniques for shape and force estimation.

## Data Availability

The raw data supporting the conclusion of this article will be made available by the authors, without undue reservation.
